# Stroke prevention in patients with acute ischemic stroke and atrial fibrillation in Germany - a cross sectional survey

**DOI:** 10.1186/s12883-019-1249-y

**Published:** 2019-02-12

**Authors:** Alexander Wutzler, Christos Krogias, Anna Grau, Roland Veltkamp, Peter U. Heuschmann, Karl Georg Haeusler

**Affiliations:** 1 Cardiovascular Center, St. Josef Hospital, University Hospital of the Ruhr-University Bochum, Bochum, Germany; 2 Department of Neurology, St. Josef Hospital, University Hospital of the Ruhr-University Bochum, Bochum, Germany; 3 Institute of Clinical Epidemiology and Biometry, University Würzburg, Würzburg, Germany; 4 Department of Stroke Medicine, Department of Neurology, Alfried Krupp Krankenhaus, Essen, Germany & Department of Neurology, Imperial College London UK, University Hospital Heidelberg, Heidelberg, Germany; 5 Comprehensive Heart Failure Center, University of Würzburg & Clinical Trial Centre Würzburg, University Hospital Würzburg, Würzburg, Germany; 6 Department of Neurology, University Hospital Würzburg, Josef-Schneider-Str. 11, 97080 Würzburg, Germany

**Keywords:** Ischemic stroke, Secondary stroke prevention, Atrial fibrillation, Survey, Oral anticoagulation, Stroke unit

## Abstract

**Background:**

Atrial fibrillation (AF) is present in 15–20% of patients with acute ischemic stroke. Oral anticoagulation reduces the risk of AF-related recurrent stroke but clinical guideline recommendations are rather vague regarding its use in the acute phase of stroke. We aimed to assess the current clinical practice of medical stroke prevention in AF patients during the acute phase of ischemic stroke.

**Methods:**

In April 2017, a standardized anonymous questionnaire was sent to clinical leads of all 298 certified stroke units in Germany.

**Results:**

Overall, 154 stroke unit leads participated (response rate 52%). Anticoagulation in the acute phase of stroke is considered feasible in more than 90% of AF patients with ischemic stroke. Clinicians assume that about two thirds of all AF patients (range 20–100%) are discharged on oral anticoagulation. According to local preferences, acetylsalicylic acid is given orally in the majority of patients with delayed initiation of oral anticoagulation. A non-vitamin K-dependent oral anticoagulant (NOAC) is more often prescribed than a vitamin K-dependent oral anticoagulant (VKA). VKA is more often chosen in patients with previous VKA intake than in VKA naive patients. In the minority of patients, stroke unit leads discuss the prescription of a specific oral anticoagulant with the treating general practitioner. Adherence to medical stroke prevention after hospital discharge is not assessed on a regular basis in any patient by the majority of participating stroke centers.

**Conclusions:**

Early secondary stroke prevention in AF patients in German stroke units is based on OAC use but prescription modalities vary in clinical practice.

**Electronic supplementary material:**

The online version of this article (10.1186/s12883-019-1249-y) contains supplementary material, which is available to authorized users.

## Background

Besides acute treatment and prevention of stroke-related complications, stroke unit treatment aims to determine stroke etiology and to optimize secondary stroke prevention. In about 15–20% of all patients with acute ischemic stroke, atrial fibrillation (AF) is either known before admission or a first episode of AF is detected in hospital [1]. In AF patients, oral anticoagulation significantly reduces the risk of recurrent stroke and is strongly recommended in AF patients after stroke [2]. Four phase III studies have demonstrated that the non-vitamin K-dependent oral anticoagulants (NOACs) are at least non-inferior compared to the well-established vitamin K antagonist (VKA) warfarin [3]. However, the safety of oral anticoagulation (OAC) given within days after acute ischemic stroke in patients with AF is unproven. Therefore, we aimed to assess the current clinical practice of medical stroke prevention on certified stroke units in Germany.

## Methods

We conducted an anonymous cross sectional survey using a standardized questionnaire (Additional file 1). A validation of the content of the questionnaire was achieved by multiple circulation and discussion in the authors group. Afterwards, formal criteria of the questionnaire were tested at the Institute of Clinical Epidemiology and Biometry, University Würzburg, Germany on the basis of pre-defined criteria. Postal dispatch, coding and evaluation of the questionnaire were carried out according to standardized procedures at the Institute of Clinical Epidemiology and Biometry.

The questionnaire was sent to clinical leads of all 298 stroke units certified by the German Stroke Society as of February 1st, 2017. Data collection was completely anonymous; no individual patient level data were collected as well as no potential personal identifiable data from the clinical leads. Thus, no specific ethics board approval was needed according to local regulations confirmed by the Ethic Committee of the Medical Faculty of the University of Würzburg. Statistical analyses were performed with SAS (Version 9.4, SAS Institute Inc.). Frequencies are reported as n (%). Assumptions on in-hospital use of oral anticoagulation or platelet inhibitors are expressed as mean (range). *P*-values < 0.05 were considered as statistically significant.

## Results

In total, 154 (52%) of the 298 stroke unit leads participated (response rate 52%). The characteristics of the responding stroke units are shown in Table 1. In accordance with the defined criteria [4], stroke unit certification level (regional vs. supraregional) was related to the number of beds at the stroke unit and to the number of stroke patients treated per year. Most of the supraregional stroke units (81%) treated > 750 stroke patients per year, while most of the regional stroke units (57%) treated ≤750 stroke patients per year. The chi-square test showed that this difference is statistically significant (*p* < 0.0001).

**Table 1:** Baseline characteristics of 154 responding German stroke centers

	Regional stroke unit	Supraregional stroke unit	Total
Number of stroke unit beds			
≤ 4	20	0	20
5–6	27	12	39
7–8	24	11	35
9–12	11	23	34
> 12	2	20	22
Total	84*	66	150***
Stroke patients treated per year			
≤ 500	17	3	20
501–750	31	9	40
751–1000	23	17	40
1001–1250	9	11	20
> 1250	4	25	29
Total	84*	65*	149***

*^/^***number of missing responses

### In-hospital use of oral anticoagulation or platelet inhibitors

Responding stroke unit leads assume that 67% (range 20–100%) of all AF patients receive OAC at hospital discharge. While 27% (range 0–80%) of all patients are discharged with a recommendation to start OAC later, OAC is assumed not to be feasible in 7% (range 0–30%) of all AF patients. If OAC is considered feasible but intake is delayed because of feared bleeding complications in the acute phase of stroke, acetylsalicylic acid (ASA) is used in most AF patients. Almost 60% of all stroke unit leads start ASA in more than 95% of these patients, while 22% do not start ASA in more than 50% of patients. If started, 100 mg ASA is given orally once daily - as stated by 99% of all stroke unit leads - and stopped more than two days before (5%), the day before (78%) or at the day of first OAC intake (17%), respectively.

### In-hospital prescription of a specific oral anticoagulant

In stroke patients considered eligible for OAC and naive regarding VKA intake, a VKA is started in more than 50% of all patients by 5% of all stroke unit leads, in 26–50% by 6%, 1–25% by 70% and in none of the patients by 19% of all stroke unit leads. In stroke patients considered eligible for OAC and previous VKA intake, a VKA is continued or reinitiated in more than 50% of all patients by 18% of all stroke unit leads, in 26–50% by 30%, 1–25% by 46% and in none of the patients by 6% of all stroke unit leads. Only 3% of all stroke unit leads prescribe one specific NOAC, while 30% chose from two NOACs, 46% prescribe one out of three and 39% prescribe one out of all four approved NOACs.

Main reasons for the prescription of a specific OAC are the individual cardiovascular risk profile (mentioned by 77% of all responding stroke unit leads), the availability of an antidote (68%), consultation with the general practitioner (58%), starting an OAC in an OAC naive patient (47%), required dosing frequency of the OAC (47%), a first episode of AF after the index stroke (42%) and net clinical benefit of the NOAC (versus VKA) according to the statement of the responsible National Association of Statutory Health Insurance Funds regulating reimbursement in Germany (23%). While 13% of all stroke unit leads report to discuss the prescription of a specific OAC with the patient’s general practitioner in more than 50% of all patients, 18% discuss specific OAC prescription in 26–50% of all patients, 57% discuss the prescription in 1–25% of all patients and 12% do not discuss their prescription. After hospital discharge, persistence regarding the prescribed OAC is not assessed in any patient by 52% of all stroke units, in 1–25% of all patients in 43%, in > 26% in 5% of all stroke units. While 26% of the responding regional stroke unit leads continue VKA treatment in > 50% of patients with previous VKA intake, only 8% of the responding supraregional stroke unit leads continue VKA in > 50% of patients pre-treated with VKA. According to the chi-square test, this difference was statistically significant (*p* < 0.004).

## Discussion

According to this cross sectional survey, most surviving stroke patients with AF are discharged on OAC and stroke unit leads consider OAC feasible in more than 90% of AF patients with ischemic stroke. However, preferences of German stroke unit leads for early secondary stroke prevention in AF patients vary to a certain extent. This reflects the missing evidence from randomized controlled trials for the acute phase of stroke and subsequent rather vague recommendations in national and international clinical guidelines [2, 5, 6].

If long-term OAC is considered feasible but prescription is delayed in the acute phase of stroke because of feared bleeding complications, a standard dose of ASA is given orally - and stopped on the day of OAC prescription - in the majority but by far not in all stroke patients with AF. This reflects the benefit of ASA in early stroke prevention on the one hand [5]; and the concerns regarding bleeding risk of OAC on the other hand [2, 6, 7]. Of note, in patients with stroke related to AF the risk of recurrent stroke is highest during the first days after stroke, with estimated event rates of 0.1–1.3% per day [8, 9]. Recently published data from the prospective observational multicenter RAF-NOAC study indicate that the time-point of NOAC initiation after ischemic stroke may impact on stroke recurrence rate and bleeding risk [10]. Nonetheless, due to the non-randomized nature of these data, there is still no profound evidence regarding the optimal handling of anticoagulation in stroke patients with AF.

Compared to VKA naive patients, a VKA is more often prescribed in AF patients with VKA intake before stroke. Interestingly, about 60% of all responding German stroke unit leads do not prescribe all four available NOACs (dabigatran, rivaroxaban, apixaban and edoxaban). The main reason for chosing a specific OAC was the individual cardiovascular risk profile, while first prescription of OAC or a first episode of AF detected after the index stroke [2] were less often mentioned. The availability of an antidote was more often mentioned compared to needed daily dosing frequency of the OAC [2, 11]. Stroke unit certification level [5] had an impact on VKA use, as leads of supraregional stroke units continue VKA after stroke significantly less frequently. Interaction between inpatient and outpatient care is considered pivotal for the success of long-term prevention [12, 13]. However, only a small minority of stroke unit leads discuss the prescription of a specific OAC with the general practitioner. Of note, a minority of stroke units routinely assess medical adherence after hospital discharge.

A limitation of our survey is that the results are based on estimations by the responders rather than on individual patient data. The response rate to our survey of more than 50% supports a representative picture of the current secondary stroke prevention in AF across German stroke units [4]. However, translation of our results to other health care settings is limited.

## Conclusion

Early secondary stroke prevention in AF patients in German stroke units is based on OAC use but prescription modalities vary in clinical practice, as demonstrated by the results of this survey. Future prospective studies in stroke patients with AF should focus on clinical questions in early secondary stroke prevention addressed in the present survey.

## Additional file

Additional file 1: Translated standardized questionnaire used during this study. Standardized anonymous German questionnaire which was sent to all clinical leads of a certified stroke unit in Germany (PDF 258 kb)

## Abbreviations

AF: Atrial fibrillation
ASA: Acetylsalicylic acid
NOAC: Non-vitamin K-dependent oral anticoagulant
OAC: Oral anticoagulation
VKA: Vitamin K-dependent oral anticoagulant

## References

[1] Rizos T, Horstmann S, Dittgen F (2016). Preexisting heart disease underlies newly diagnosed atrial fibrillation after acute ischemic stroke. Stroke.

[2] Kirchhof P, Benussi S, Kotecha D (2016). 2016 ESC guidelines for the management of atrial fibrillation. Eur Heart J.

[3] Ruff CT, Giugliano RP, Braunwald E (2014). Comparison of the efficacy and safety of new oral anticoagulants with warfarin in patients with atrial fibrillation: a meta-analysis of randomised trials. Lancet.

[4] Nabavi DG, Ossenbrink M, Schinkel M (2015). Revised certification criteria for regional and national stroke units in Germany. Nervenarzt.

[5] Kernan WN, Ovbiagele B, Black HR (2014). Guidelines for the prevention of stroke in patients with stroke and transient ischemic attack. Stroke.

[6] Powers WJ, Rabinstein AA, Ackerson T (2018). 2018 guidelines for the early Management of Patients with Acute Ischemic Stroke: a guideline for healthcare professionals from the American Heart Association/American Stroke Association. Stroke.

[7] Rothwell PM, Algra A, Chen Z (2016). Effects of aspirin on risk and severity of early recurrent stroke after transient ischaemic attack and ischaemic stroke: time-course analysis of randomised trials. Lancet.

[8] Paciaroni M, Agnelli G, Falocci N (2015). Early recurrence and cerebral bleeding in patients with acute ischemic stroke and atrial fibrillation: effect of anticoagulation and its timing: the RAF study. Stroke.

[9] Abdul-Rahim AH, Fulton RL, Frank B (2015). Association of improved outcome in acute ischemic stroke with atrial fibrillation who receive early antithrombotic therapy: analysis from VISTA. Eur J Neurol.

[10] Paciaroni M, Agnelli G, Falocci N (2017). Early recurrence and major bleeding in patients with acute ischemic stroke and atrial fibrillation treated with non-vitamin-K Oral anticoagulants (RAF-NOACs) study. J Am Heart Assoc.

[11] Steffel J, Verhamme P, Potpara TS (2018). The 2018 European heart rhythm association practical guide on the use of non-vitamin K antagonist oral anticoagulants in patients with atrial fibrillation. Eur Heart J.

[12] Kirchhof P, Breithardt G, Bax J (2016). A roadmap to improve the quality of atrial fibrillation management: proceedings from the fifth atrial fibrillation network/European heart rhythm association consensus conference. Europace.

[13] Haeusler KG, Gerth A, Limbourg T (2015). Use of vitamin K antagonists for secondary stroke prevention depends on the treating healthcare provider in Germany - results from the German AFNET registry. BMC Neurol.

