# Supplementary material for: Stroke prevention in patients with acute ischemic stroke and atrial fibrillation in Germany - a cross sectional survey
Source: BMC Neurol. 2019 Feb 12;19:25. doi: 10.1186/s12883-019-1249-y (PMC6371606; doi:10.1186/s12883-019-1249-y)
Supplement: Supplementary file 1 — Translated standardized questionnaire used during this study. Standardized anonymous German questionnaire which was sent to all clinical leads of a certified stroke unit in Germany (PDF 258 kb) [file 12883_2019_1249_MOESM1_ESM.pdf]

Questionnaire for certified stroke units : “Stroke prevention in patients with acute ischemic stroke and atrial fibrillation in Germany“

Alexander Wutzler[1], Christos Krogias[2], Roland Veltkamp[3], Peter U. Heuschmann[4], Anna Grau[4], Karl Georg Häusler[5]

[1] Rhythmologische Abteilung, Klinik für Kardiologie der Ruhr-Universität Bochum, St. Josef-Hospital

[2] Department of Neurology, Ruhr-Universität Bochum, St. Josef-Hospital Bochum

[3] Imperial College London, UK

[4] Institute of Clinical Epidemiology and Biometry, Julius Maximilian Universität Würzburg

[5] Department of Neurology & Center for Stroke Research Berlin, Charité - Universitätsmedizin Berlin

Dear colleague,

Thank you very much for taking the time to participate in our survey “Stroke prevention in patients with acute ischemic stroke and atrial fibrillation in Germany”. Completing the questionnaire will last approximately 10 minutes.

Please answer all questions

- Please mark the questionnaire clearly using a black or blue ballpoint pen.
- Please note the instructions permitted for each question.
- Please tick boxes clearly [X]

Please send the completed questionnaire to:

Julius Maximilian Universität Würzburg
Institute for Clinical Epidemiology and Biometry
c/o. Anna Grau
Josef-Schneider-Str. 2 / Haus D7
97080 Wuerzburg

You can use the enclosed prepaid and addressed envelope. Please do not provide any address of the sender.

In case of any question, do not hesitate to contact us:

Dr. Karl Georg Häusler: Email: Georg.Haeusler@charite.de
Anna Grau: Email: Grau_A@ukw.de

Thank you very much for your support and your time!

**Please specify the number of ischemic stroke patients treated at your institution in 2016. Please also specify the number of stroke unit beds and the level of stroke unit certification at your institution.**<br>*Note: Single response per column, please estimate.*					
**Number of ischemic stroke patients treated in 2016?**		**Number of stroke unit beds in 2016?**		**Level of certification in 2016?**	
A) <= 500		A) <= 4		A) regional	
B) 501 – 750		B) 5 - 6		B) superregional	
C) 751 -1,000		C) 7 - 8			
D) 1,001 - 1,250		D) 9 - 12			
E) > 1250		E) > 12			

**1. What is the percentage of ischemic stroke patients in whom atrial fibrillation is either diagnosed before or during the in-hospital stay?**<br>*Note: Single response per column, please estimate.*			
**Known atrial fibrillation <u>before hospital admission because of the index stroke</u>**		**<u>First episode of atrial fibrillation during the in-hospital stay after the index stroke</u>**	
A) 0 - 5 %		A) 0 - 5 %	
B) 6 - 10 %		B) 6 - 10 %	
C) 11 - 20 %		C) 11 - 20 %	
D) > 20 %		D) > 20 %	

**2. What is the percentage of ischemic stroke patients with diagnosed atrial fibrillation in whom an echocardiography is performed during the in-hospital stay?**<br>*Note: Single response per column, please estimate.*					
**Transthoracic echocardiography only**		**Transesophageal echocardiography only**		**Transesophageal and transthoracic echocardiography**	
A) 0 %		A) 0 %		A) 0 %	
B) 1 - 20 %		B) 1 - 20 %		B) 1 - 20 %	
C) 21 - 40 %		C) 21 - 40 %		C) 21 - 40 %	
D) > 40 %		D) > 40 %		D) > 40 %	

**3. If an echocardiography is performed in an ischemic stroke patient with diagnosed atrial fibrillation: Which question(s) do you want to address by performing an echocardiography?**<br>*Note: Multiple responses are possible.*	
A) Possible diagnosis of left atrial thrombi	
B) Possible diagnosis of concurrent causes of the ischemic stroke (e.g: left atrial thrombus, aortic plaques)	
C) Possible diagnosis of a cardiac failure	
D) Possible differentiation between valvular and non-valvular atrial fibrillation	

**4. In which percentage of your ischemic stroke patients with diagnosed non-valvular atrial fibrillation do you either start treatment with oral anticoagulation before hospital discharge or recommend starting oral anticoagulation after hospital discharge or consider oral anticoagulation as not feasible?**<br>*Note: Please estimate.*	
Start of oral anticoagulation before hospital discharge:	
Recommendation to start oral anticoagulation after hospital discharge:	
No recommendation given to start oral anticoagulation:	
**Sum:**	**1 0 0 %**

**5. If you consider oral coagulation to be feasible in an ischemic stroke patient with atrial fibrillation but start of treatment is delayed due to the feared risk of bleeding: In which percentage of your ischemic stroke patients with diagnosed non-valvular atrial fibrillation do you <u>NOT</u> prescribe acetylsalicylic acid as early secondary prevention?**<br>*Note: Single response, please estimate.*	
A) < 5 %	
B) 5 - 25 %	
C) 26 - 50 %	
D) > 50 %	

6. If, in principle, you consider oral anticoagulation to be feasible in stroke patients with known non-valvular atrial fibrillation, but you do not initiate oral anticoagulation immediately after the acute ischemic event due to the assumed cerebral bleeding risk but instead prefer giving acetylsalicylic acid (ASA) for early secondary prevention:

Note: Single response per column, please estimate.

**How do you usually prescribe acetylsalicylic acid in stroke patient with atrial fibrillation:**		**Until which time before the planned start of NOAC administration do you continue to give acetylsalicylic acid in stroke patients with known atrial fibrillation?**	
A) 100 mg OD orally		A) The last dose is administered on the first day of NOAC administration	
B) 300 mg OD orally		B) The last dose is administered on the day before the first NOAC administration	
C) 100 - 250 mg OD i.v.		C) The last dose is administered 2-4 days before the first NOAC administration	
D) 300 - 500 mg OD i.v.		D) The last dose is administered >5 days before the first NOAC administration	

7. If you consider oral anticoagulation to be feasible in stroke patients with known non-valvular atrial fibrillation as secondary prevention:
For which percentage of stroke patients do you prescribe administration of a Vitamin K antagonist (VKA) during hospital care, or recommend prescription of a VKA after hospital discharge

Note: Single response per column, please estimate.

**In stroke patients <u>without previous</u> VKA intake:**		**In patients <u>with previous</u> VKA intake:**	
A) 0 %		A) 0 %	
B) 1 - 25 %		B) 1 - 25 %	
C) 26 - 50 %		C) 26 - 50 %	
D) > 50 %		D) > 50 %	

**8. If you consider oral anticoagulation to be feasible as secondary prevention in patients with known non-valvular atrial fibrillation and you decide to prescribe NOAC during hospital care: How many different NOACs do you use for your patients?**<br>*Note: Single response, please estimate.*	
A) one of the four approved NOACs	
B) two of the four approved NOACs	
C) three of the four approved NOACs	
D) all of the four approved NOACs	

**9. If you start oral anticoagulation for secondary prevention in stroke patients with known non-valvular atrial fibrillation with a particular oral anticoagulant, or you recommend a particular oral anticoagulant after inpatient treatment: For which proportion of patients do you discuss the prescription or recommendation of a particular NOAC in advance with the next ambulatory care provider?**<br>*Note: Single response, please estimate.*	
A) 0 %	
B) 1 - 25 %	
C) 26 - 50 %	
D) > 50 %	

**10. If you start oral anticoagulation for secondary prevention in stroke patients with known non-valvular atrial fibrillation or you recommend initiation after inpatient hospital treatment: In which proportion of your stroke patients do you check whether the patient continues to take the prescribed oral anticoagulant after inpatient care?**<br>*Note: Single response, please estimate.*	
A) 0 %	
B) 1 - 25 %	
C) 26 - 50 %	
D) > 50 %	

**11. If you prescribe oral anticoagulation for secondary prevention in stroke patients with known non-valvular atrial fibrillation during inpatient hospital treatment or you recommend prescription after the inpatient period: Which aspects, among others, influence your selection of the particular drug?**<br>*Note: Multiple responses are possible.*	
A) Individual (cardiovascular) risk profile of the stroke patient.	
B) Frequency of necessary daily administration of the drug.	
C) Availability of an antidote for the drug.	
D) Existing positive evaluation of the product according to the German "Pharmaceuticals Market Reorganisation law (AMNOG)".	
E) Result of previous consultation with the ambulatory care providers.	
F) First-time administration of an oral anticoagulation for a patient with non-valvular atrial fibrillation.	
G) First-time detection of non-valvular atrial fibrillation during the hospitalization of the patient due to ischemic stroke.	
